# Supplementary material for: Electrically insulating PBO/MXene film with superior thermal conductivity, mechanical properties, thermal stability, and flame retardancy
Source: Nat Commun. 2023 Sep 2;14:5342. doi: 10.1038/s41467-023-40707-x (PMC10475028; doi:10.1038/s41467-023-40707-x)
Supplement: Supplementary file 1 — Supplementary Information.pdf [file 41467_2023_40707_MOESM1_ESM.pdf]

Supplementary Information

Electrically insulating PBO/MXene film with superior thermal conductivity, mechanical properties, thermal stability, and flame retardancy

Yong Liu[1,2], Weizhi Zou[1,2], Ning Zhao[1,2,*], Jian Xu[1]

[1] Beijing National Laboratory for Molecular Sciences, Laboratory of Polymer Physics and Chemistry, Institute of Chemistry, Chinese Academy of Sciences, Beijing 100190, P. R. China

[2] University of Chinese Academy of Sciences, Beijing 100049, P. R. China

*Corresponding Author. Email address: zhaoning@iccas.ac.cn (N. Zhao)

Supplementary Methods

Material. MAX (Ti_3AlC_2) (200 Meshes, 98%) and phenol were bought from Shanghai Macklin Biochemical Co., Ltd. PBO fibre was obtained from the Zhongke Jinqi New Material Technology Co., Ltd. (Zhejiang, China). Hydrochloric acid (HCl, 37 wt%) was got from Sinopharm Chemical Reagent Co., Ltd. (Shanghai, China). Lithium fluoride (LiF, AR, 98.5%) was bought from Alfa Aesar Chemicals Co., Ltd. (Shanghai, China). Ethyl acetate (EA, GR) and isopropanol (IPA, GR) were got from Concord Technology Co., Ltd. (Tianjin, China). Methane sulfonic acid (MSA, 99%) and trifluoroacetic acid (TFA, 99.9%) were obtained from J＆K Scientific Co., Ltd. (Beijing, China). All chemicals are used directly.

Fabrication of $Ti_3C_2T_x$ MXene nanosheets and PBO nanofibres. MXene nanosheets were prepared according to the method reported elsewhere[1]. Briefly, 3.2 g LiF was dissolved into 40 mL HCl solution (9 M) in a polypropylene plastic bottle, and 2 g MAX powders were added slowly within 10 min. The mixture was stirred at 35 °C in a water bath for 24 h. Then the mixture was washed with water and separated through centrifugation at 1369 x g for 5 min. This process should be repeated several times until the pH value of the mixture approached 7. Next, 30 mL ethanol was added to the precipitate and ultrasonic treatment for 60 min, and then centrifuged at 11180 x g for 10 min. The latest precipitate was mixed with 30 mL of water under ultrasonic treatment for 20 min in a nitrogen atmosphere. After centrifugation (2795 x g, 5 min), dispersion of single- or few-layer MXene could be obtained. MXene powders were obtained by freeze-drying the MXene dispersion.

PBO nanofibres were obtained by exfoliating PBO fibres[2]. In brief, PBO fibres were added into a mixture of MSA and TFA with a mass ratio of 1:1, and then stirred for 3 days at room temperature to obtain a PBO nanofibre acid sol (1 wt %).

Characterization. ATR-FTIR spectra were recorded on BRUKER TENSOR 27 FTIR spectrometer. The morphology of samples was investigated by SEM on a JEOL JSM-7500F at 5 kV and by TEM on a JEOL-JEM 2100F at 200 kV, respectively. The thickness of the MXene nanosheet was measured by AFM on a Bruker Dimension ICON. The energy dispersive

spectroscopy (EDS) on JEOL JSM 7100F was used to analyze the element distribution of the fracture nanocomposite film. The crystalline structure of the film was characterized by an X-ray diffractometer (XRD, Empyrean). The Raman spectra were recorded from 100 to 4000 cm^{-1} on a LabRAM HR Evolution using a 532 nm NeHe laser. The Anton Paar MCR 302 rheometer was used for the rheological characterization of gels. Tensile tests of the film (~ 3 mm width) were performed on a UTM-16555 (Shenzhen Suns Technology Stock Co., Ltd.) at a tensile speed of 1 mm min^{-1}. Tensile tests were carried out using the single-edge notched samples (film width of 5 mm, notched length of 1 mm, tensile speed of 1 mm min^{-1}) to investigate the fracture energy. The fracture energy was calculated according to the following equation:

$$\text{Fracture energy} = \frac{6wc}{\sqrt{\lambda_c}} \tag{1}$$

where λ_c is the fracture strain of the notched sample, c is the notch length and w is the integration of stress–strain curve until λ_c for the unnotched sample. X-ray Photoelectron Spectroscopy (XPS) measurements were carried out with a Thermo Scientific ESCALab 250Xi. The electrical conductivity of the films was measured by a high-precision four-probe instrument (MCP-T700, Measurement range: 0.001×10^{-4} ~ 9.999×10^{7} Ω). Volume resistance of the films was measured by a Keithley 6517B. Thermogravimetric analysis (TGA) was performed on a PE Pyris 1 with a heating rate of 10 °C min^{-1} from 30 to 800 °C in an air atmosphere. The ignition and flame shielding properties of films were tested using the oxygen index (JF-3A) and vertical combustion (TTech-GBT2408) according to ISO 4589-2 and ISO 9773-1998 standard, respectively. The thermal conductivity (TC) was calculated according to the following formula:

$$\text{TC} = \alpha \times \rho \times C_p \tag{2}$$

where α, ρ and C_p are the thermal diffusivity, density, and specific heat capacity of the nanocomposite film, respectively; α was measured with the laser-flash method (LFA 447, NETZSCH, Germany); ρ was obtained by weighing; and C_p was measured by DSC (TA-Q2000) using the sapphire method. Temperature changes in thermal management were recorded dynamically with the Fluke thermal imager TiS65. Two-dimensional wide-angle X-ray scattering (2D WAXS) measurements were carried out on a SmartLab X-ray diffractometer

with HyPix 3000 detector. The incident beam was almost parallel to the film face. The in-plane orientation of PBO nanofibres with and without MXene nanosheets was calculated from the azimuthal profile of (200) reflection using the following equations:

$$\text{Herman's orientation parameter } (f) = \frac{3 < \cos^2\varphi > -1}{2} \tag{3}$$

$$< \cos^2\varphi >= \frac{\sum_0^{\pi/2} \mathrm{I}(\varphi)\sin\varphi\cos\varphi}{\sum_0^{\pi/2} \mathrm{I}(\varphi)\sin\varphi} \tag{4}$$

where φ and I are the azimuthal angle and the corresponding integral intensity, respectively. Notably, φ is also the angle between the film face and the PBO nanofibres. The increase in f from 0 to 1 indicates that the nanofibres are distributed from completely random to completely parallel to the film face.

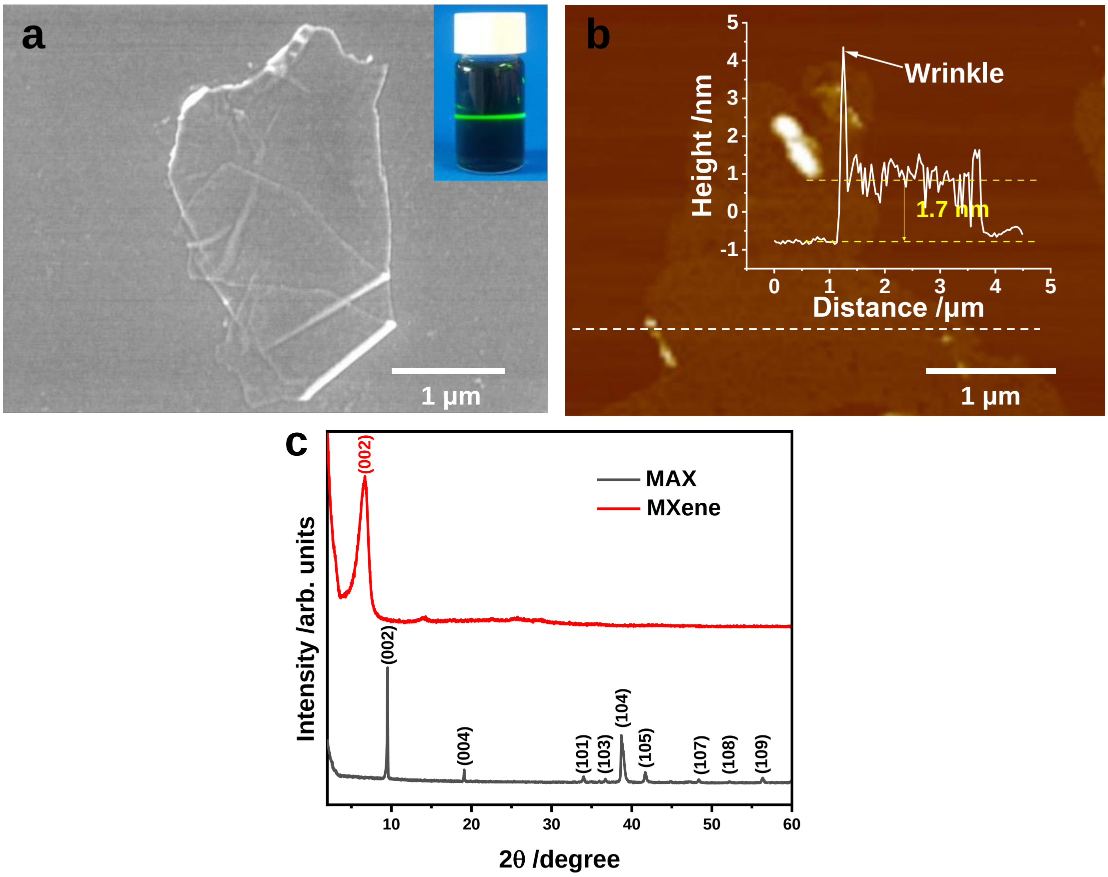

Supplementary Figure 1. Characterization of MXene nanosheets. **a** SEM and **b** AFM images of the as-formed $Ti_3C_2T_x$ MXene. **c** XRD patterns of Ti_3AlC_2 MAX and $Ti_3C_2T_x$ MXene. The inset in (**a**) displays Tindal effect of the obtained dispersion of MXene nanosheets, indicating good hydrophilicity and dispersibility.

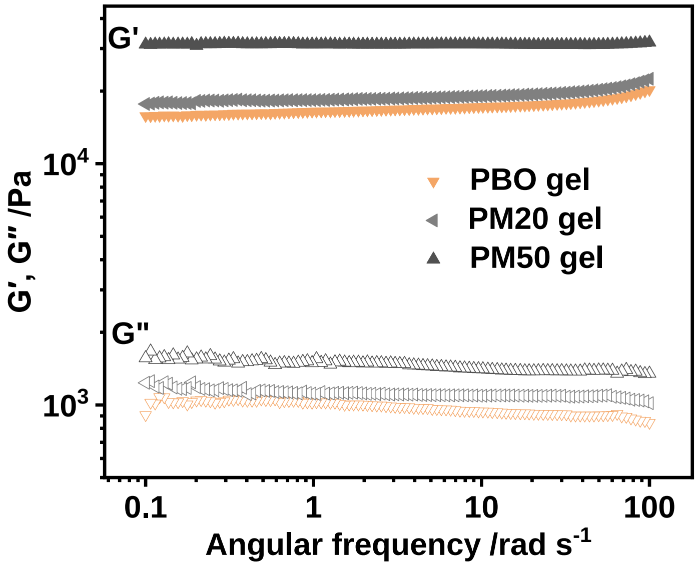

Supplementary Figure 2. The rheological properties of gels. Variation of elastic moduli (G′) and loss moduli (G″) with angular frequency for PBO, PM20 and PM50 alcohol gels. The result demonstrates the formation of the elastic networks in PBO and PBO/MXene alcohol gels. Furthermore, G' and G" of the gels increased with MXene concentration, indicating the enhancing effect of MXene on the network strength.

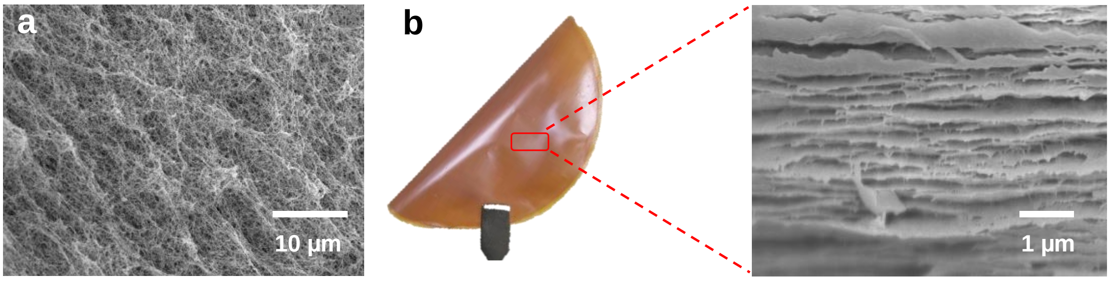

Supplementary Figure 3. Morphology of PBO gel and film. **a** SEM images of the freeze-dried PBO gel. **b** The optical photograph and cross-sectional morphology of PBO film.

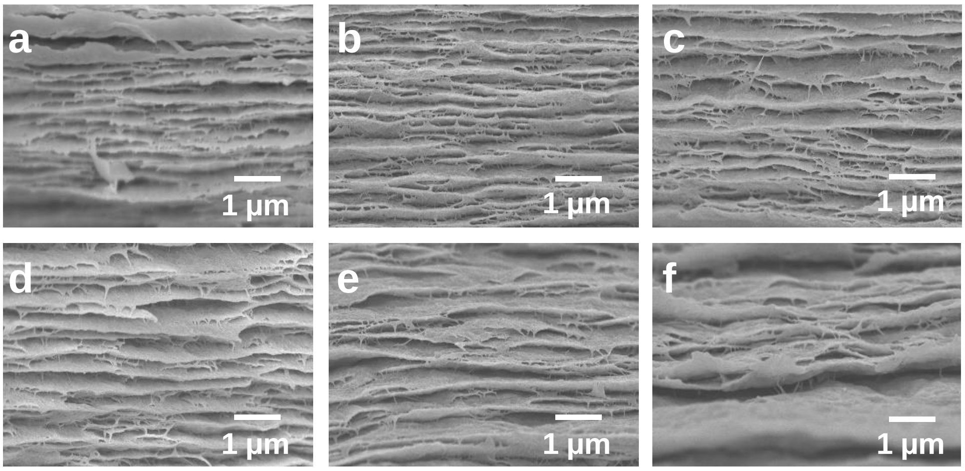

Supplementary Figure 4. The cross-section SEM images of the films. **a** PBO. **b** PM10. **c** PM20. **d** PM30. **e** PM50. **f** PM70. It can be seen that the interlayer stacking density of composite film reduces with the increase of the MXene content, indicating a decrease in the interaction between the layers.

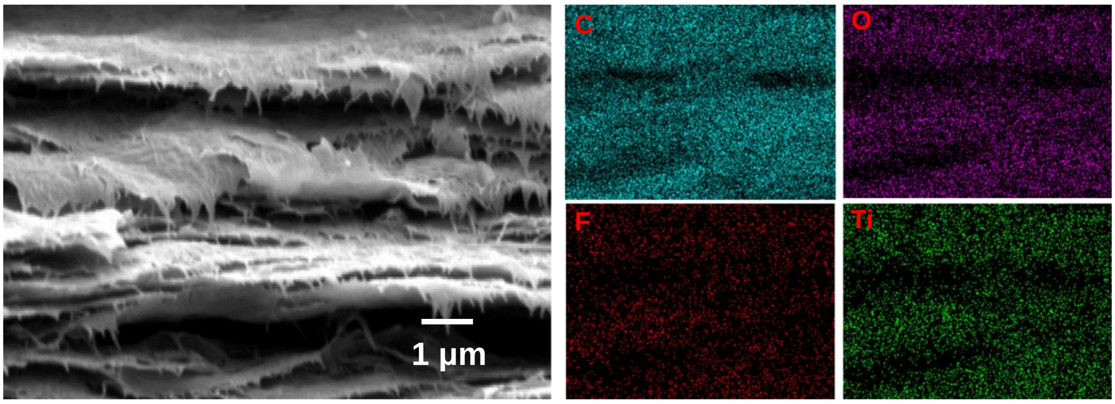

Supplementary Figure 5. Elemental mapping images of the fracture of PBO/MXene film (PM20), showing a homogeneous distribution of MXene in the laminar composite film.

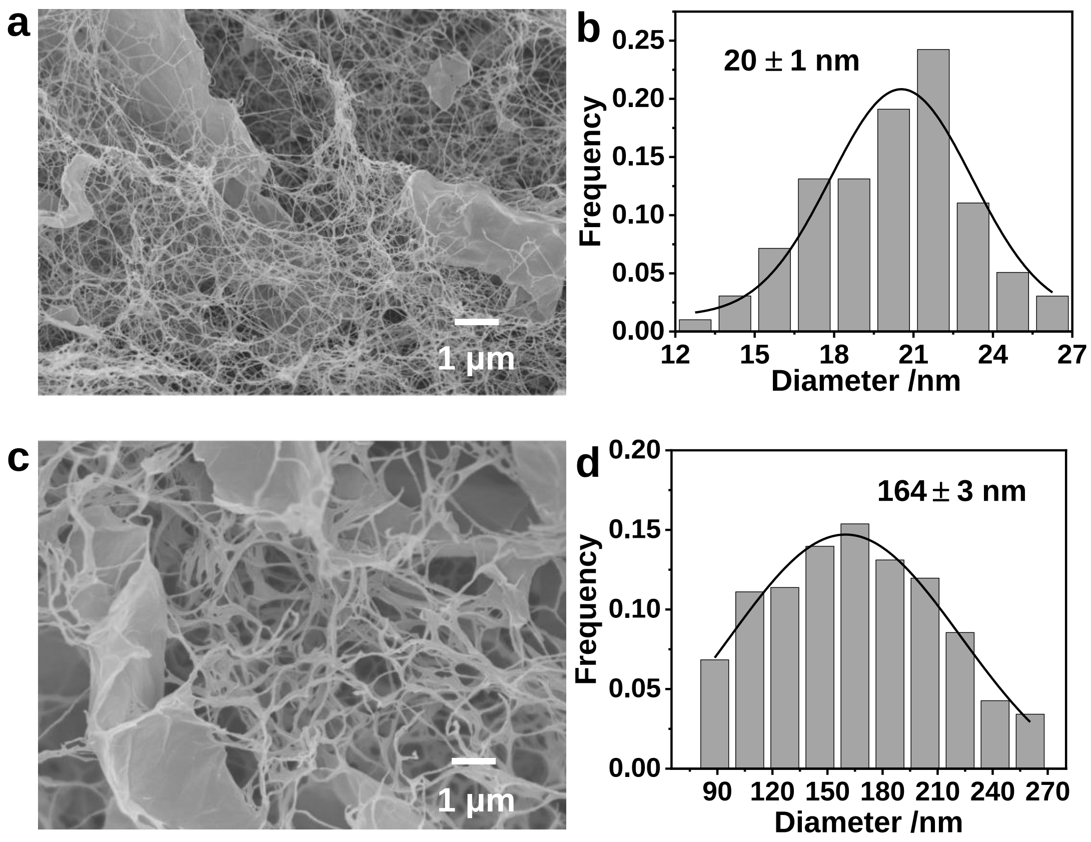

Supplementary Figure 6. Morphology of PBO/MXene gel networks. **a** SEM image of PM50 gel network prepared by proton-consumption-induced gelation and **b** size distribution of the corresponding PBO nanofibres. **c** SEM image of the control sample (W-PM50) gel network prepared by water-vapor-induced gelation and **d** size distribution of the corresponding PBO nanofibres. It can be seen that both the PBO nanofibres and MXene nanosheets show a lower degree of aggregation in the PBO/MXene gels by proton-consumption-induced gelation. Meanwhile, there is a finer and denser nanofibre network adhered to the nanosheets.

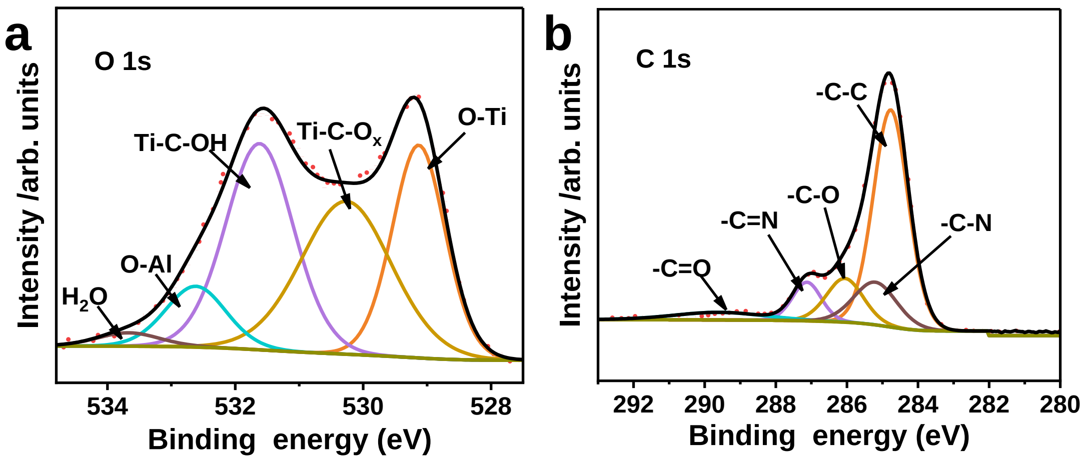

Supplementary Figure 7. XPS analysis of MXene and PBO films. **a** The high-resolution O 1s spectrum of MXene film and **b** the high-resolution C 1s spectrum of PBO film. The fitted O 1s spectra indicate the presence of the polar group Ti−C−OH on the surface of MXene nanosheets. The fitted C 1s spectra indicate the presence of the polar groups −C=O and −C=N on the surface of the PBO nanofires.

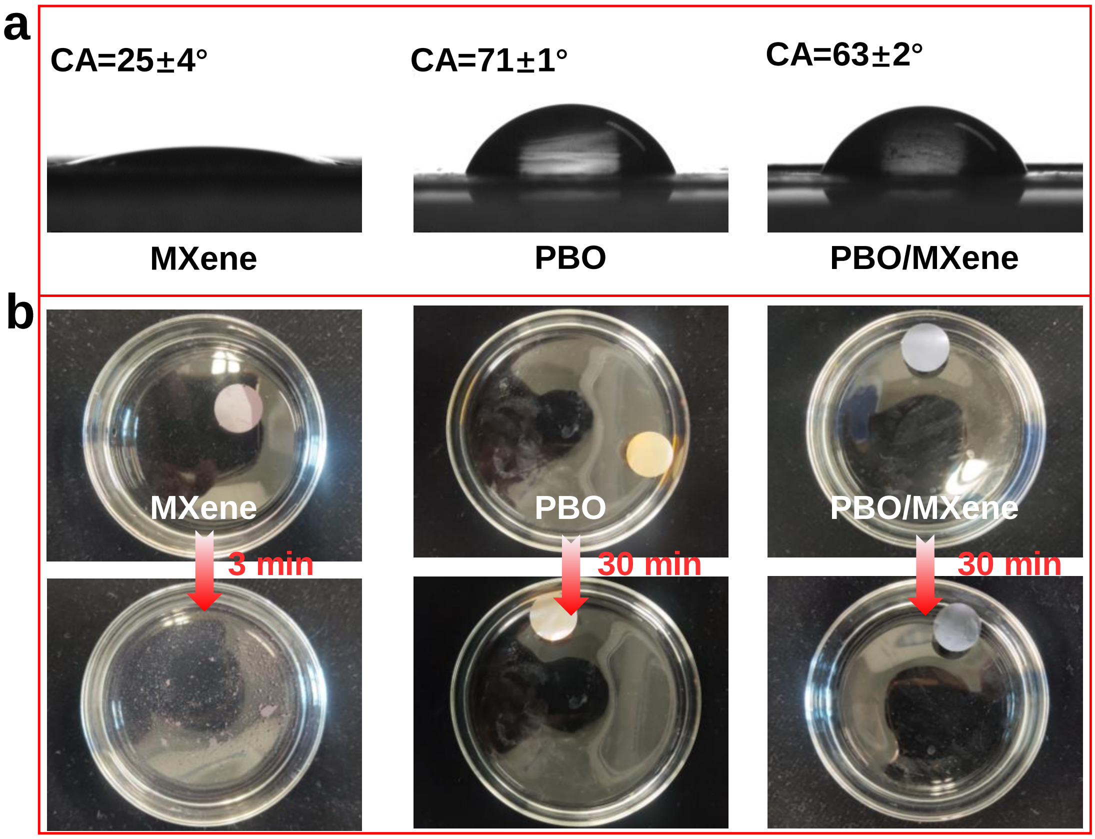

Supplementary Figure 8. Structural stability investigation of the films. **a** Water contact angle (CA) of MXene, PBO and PM20 films. **b** Optical photographs of MXene, PBO and PM20 films before and after ultrasonic treatment. PBO/MXene composite film has a similar wettability to PBO film, further confirming the hydrophilic MXene nanosheets in the composite film were caged by the relatively hydrophobic PBO nanofibre network. The caging effect and the strong interaction between PBO and MXene endow the composite film with better stability against ultrasonication than pristine MXene film. The improved hydrophobicity also protects the film backbone from being weakened by water, and mitigates the shear damage to the film caused by the cavitation bubbles generated by ultrasonication.

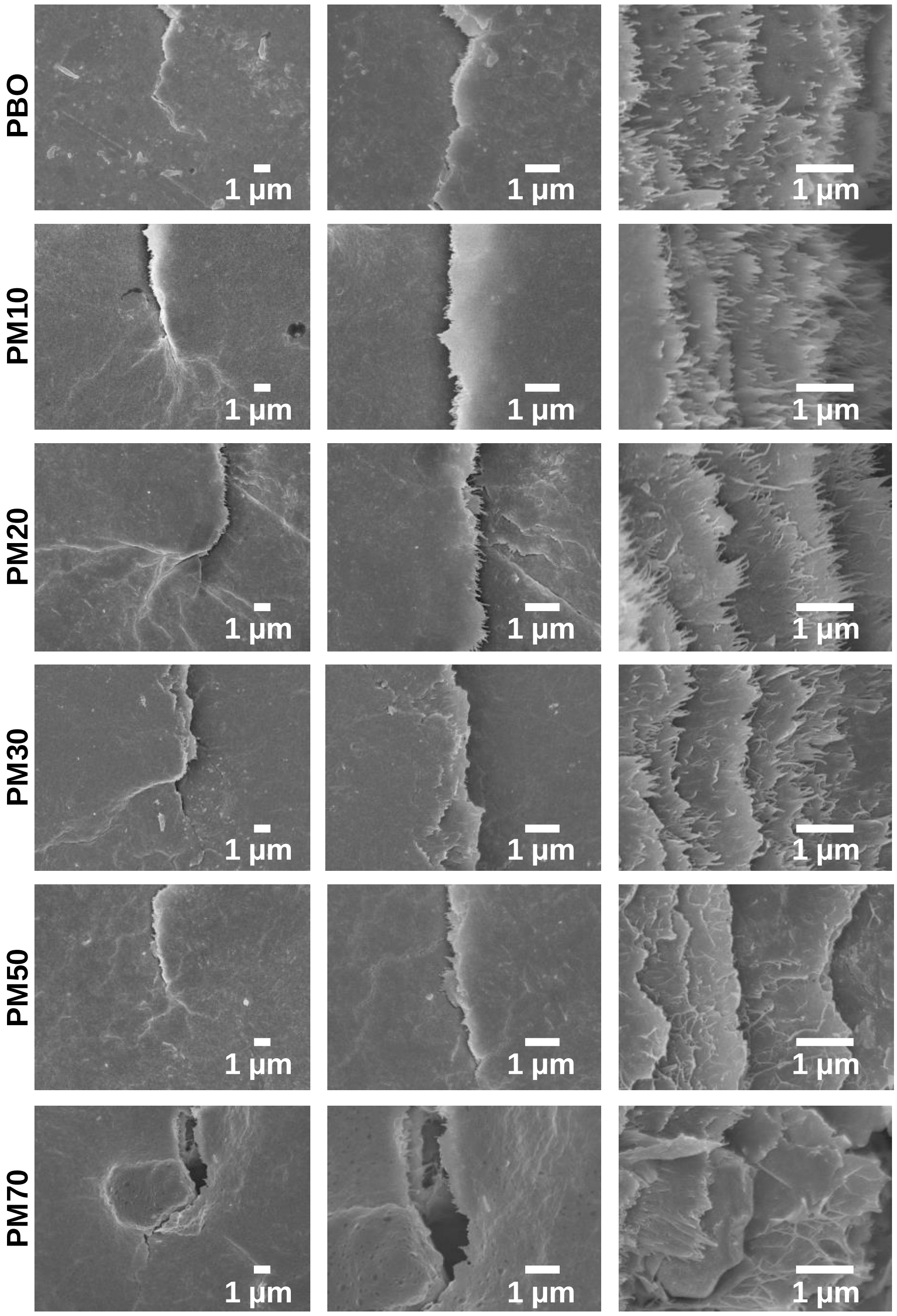

Supplementary Figure 9. SEM images of the propagated cracks for PBO/MXene films with different MXene contents.

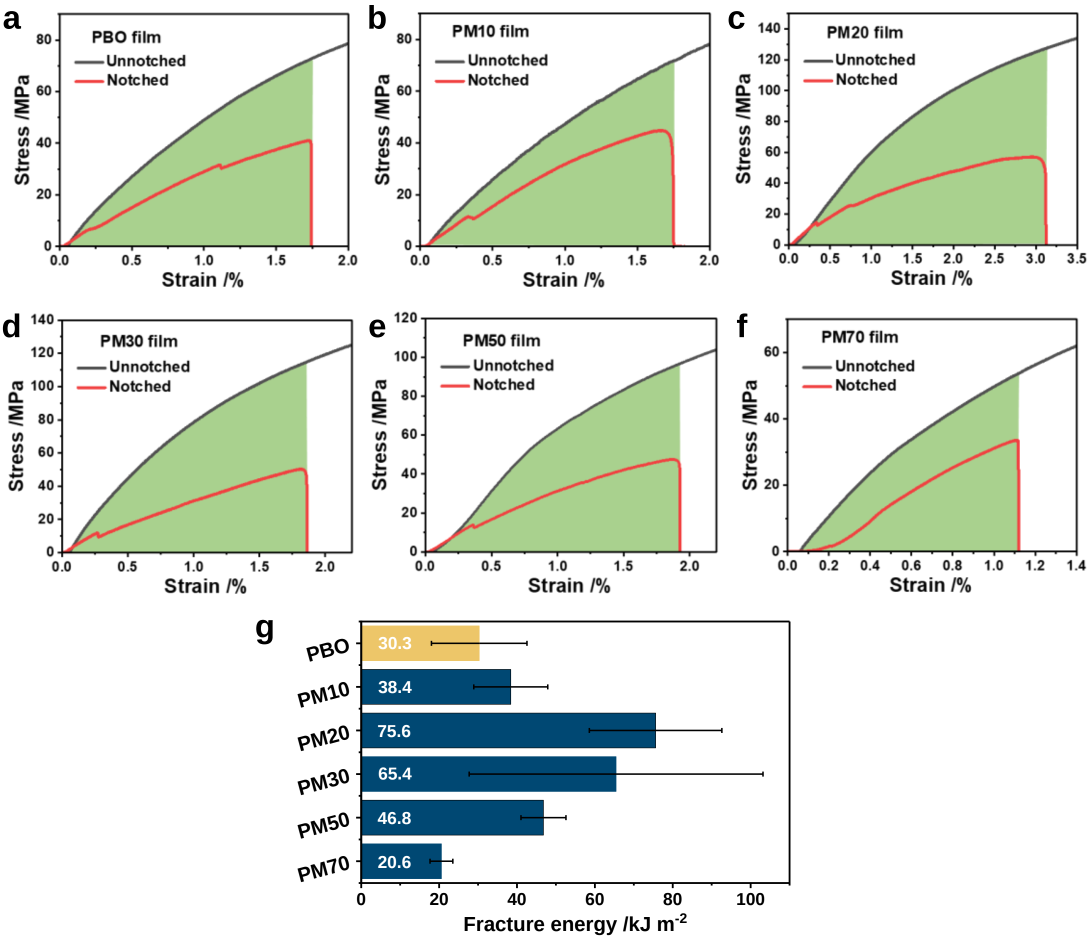

Supplementary Figure 10. Fracture energy tests of the films. **a-f** Stress–strain curves of notched samples. **g** Statistics of fracture energy. Error bars represent standard deviations (n = 3).

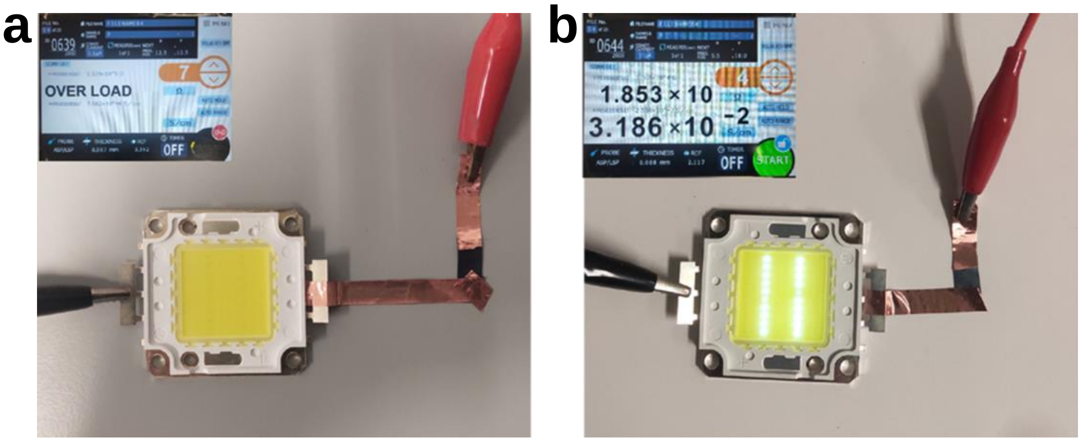

Supplementary Figure 11. The electrical conductivity tests of the films. **a** PM50. **b** W-PM50. The extremely low electrical conductivity of the PM50 is beyond the measurement range of the high-precision four-probe instrument (MCP-T700).

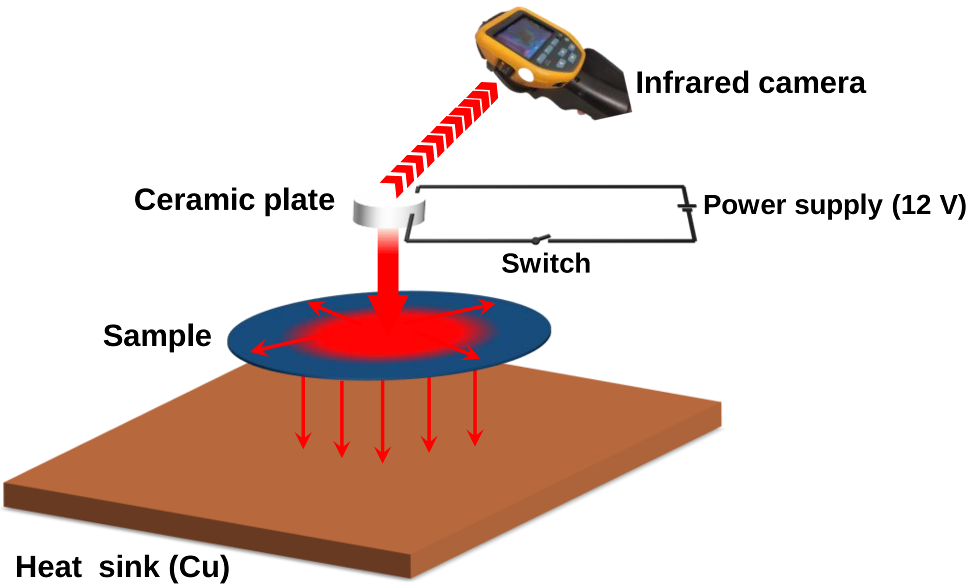

Supplementary Figure 12. The setup to demonstrate the potential of tested sample as a heat spreader for high-power ceramic plate.

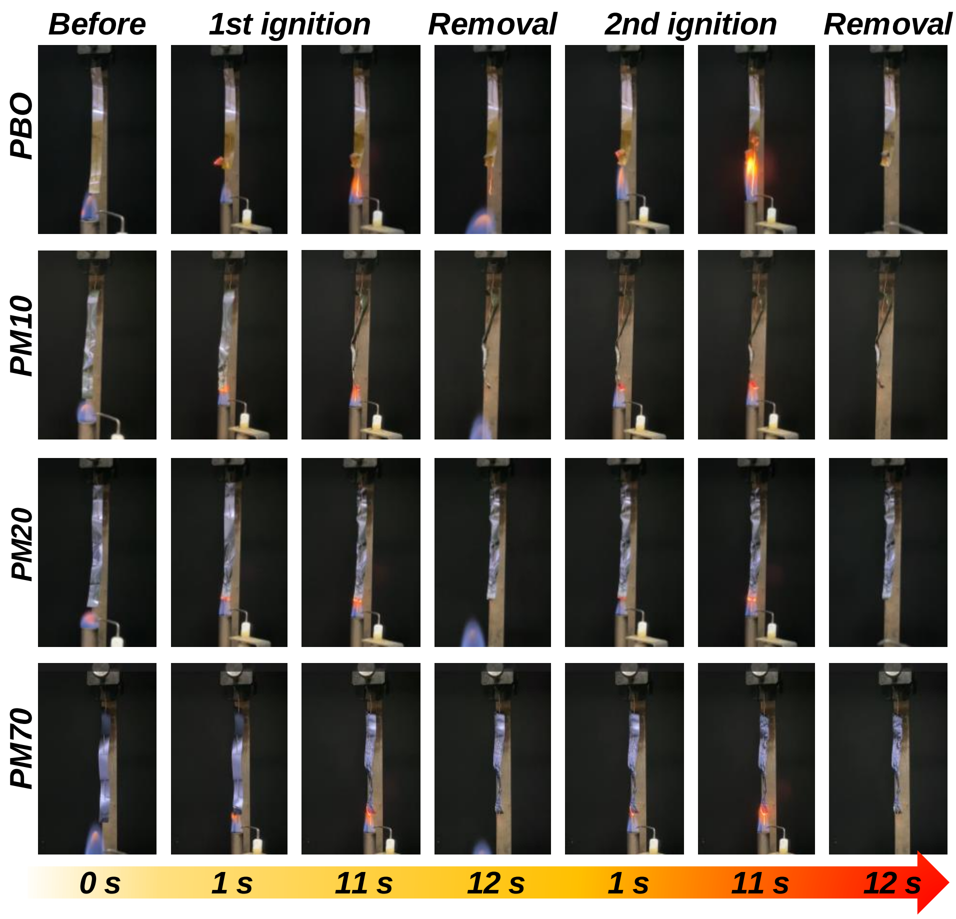

Supplementary Figure 13. Snapshots of vertical combustion tests for PBO and PBO/MXene films.

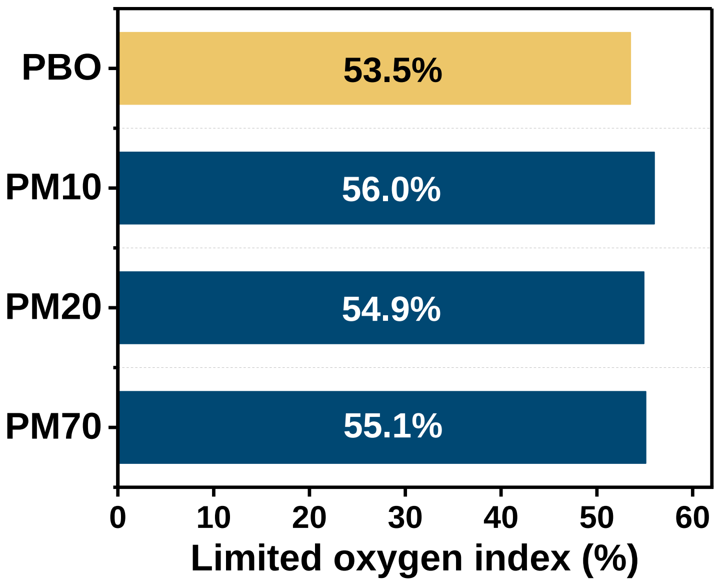

Supplementary Figure 14. Limited oxygen index of PBO and PBO/MXene films.

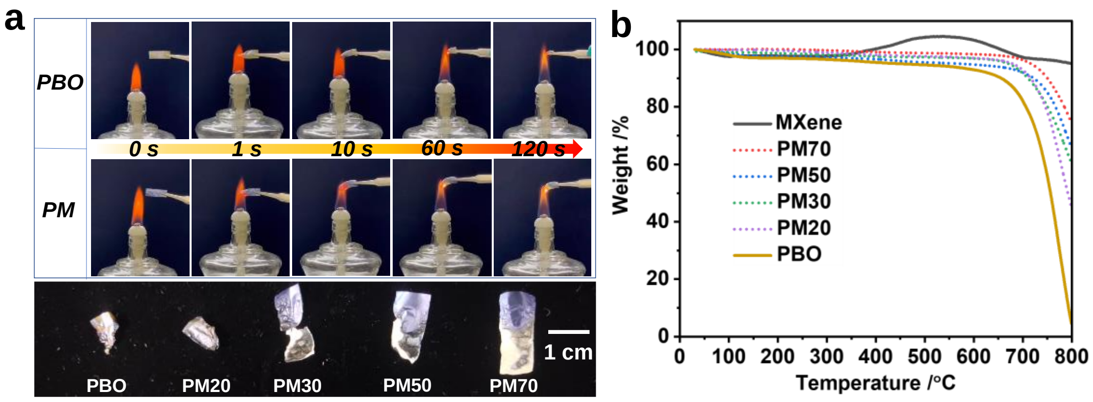

Supplementary Figure 15. Heat and fire resistance tests for films. **a** Burning of PBO and PBO/MXene films on an alcohol lamp for 120 s and their residues. **b** TGA curves of MXene, PBO and PBO/MXene films in air.

Supplementary Table 1. Properties of commercially available thermally conductive plastics.

Company	Polymer matrix	Grade	Strength (MPa)	TC (W m^{-1} K^{-1})	Electrical resistance (Ω cm)
Cool Polymers[a]	PPS	D5108	36	10	2.5×10^{16}
	PPS	D1202	25	5	$>1\times10^{9}$
	PPS	D5110	46	1.5	$>1\times10^{9}$
	LCP	D5506	55	10	1.6×10^{16}
	PPS	E5101	45	20	1.1×10^{3}
	PPS	E5105	60	4.5	$>1\times10^{9}$
	PA6	E3607	50	14	$<1\times10^{9}$
DSM[a]	PA46	Stanyl-TC502	65	14	1×10^{6}
	PA46	Stanyl-TC551	50	14	1×10^{6}
	PA46	Stanyl-TC154	55	1.0	1×10^{15}
	PA46	Stanyl-TC155	55	5.0	-
	PA46	Stanyl-TC153	55	8.0	1×10^{15}
Laticonther[a]	PA6	62GR/70	-	28	1×10^{2}
	PPS	80GR/50	60	10	2×10^{3}
	PA6	62GR/50	-	12	1×10^{4}
Mitsubishi[b]	PC	TPN2131	81	4.9	2×10^{12}
	PC	TPN2140	65	3.3	5×10^{12}
	PC	TPN1125	44	21.3	2×10^{4}
	PC	TPN1124	57	14.4	5×10^{4}
	PC	TPN1122	80	8.8	2×10^{4}
	PC	TPN1140	108	3.3	2×10^{5}
	PC	TPN1022	48	13.2	2×10^{4}
Avient[a]	PA66	NNC-5000	48.3	11	1×10^{4}
	PA12	NJC-6000	49	11	1×10^{3}
	PA12	NJC-7500	39.3	24.9	1×10^{5}
	PA12	NJC-6500	36.4	≥16.0	2.2×10^{4}

a: https://www.matweb.com.

b: https://www.m-ep.co.jp/ch/product.

Supplementary Table 2. Surface elemental content of PBO, MXene and PBO/MXene films.

Sample	C (At%)	O (At%)	N (At%)	F (At%)	Ti (At%)
PBO	75.24	15.74	9.02		
MXene	38.41	27.00	1.32	7.13	26.14
PM20	74.12	15.08	10.77	0.03	
PM50	76.05	14.05	9.66	0.18	0.06

Supplementary Table 3. Comparison of mechanical properties of nacre-inspired PBO/MXene films with those of other polymer/2D inorganic nanosheet composites.

Materials	Loading (wt%)	Tensile strength (MPa)/Young' modulus (GPa)	Toughness (MJ m^{-3})	Ref.
ANF/$Ti_3C_2T_x$	40	300/13.6	5.3	1
CNF/MXenes	50	135/3.8	14.7	3
CNF/MXenes	40	212/5.9	5.3	4
CNF/BC/MXenes	-	252.6/-	34.3	5
CMC/$Na_2B_4O_7$/MXene	-	583/27.8	15.9	6
PBONF/GNS	50	192/4	44	7
NFC/GS	15	84.5/6.19	1.29	8
ANF/GF	20	188.5/5.2	107	9
CNF/rGO	94.7	270/7.4	9.3	10
Cellulose/BNNS	50	226/6.6	15.2	11
SiO_2-coated NFC/BNNS	7	166/7.2	11	12
ANF/BNNS	30	167/6.0	6.2	13
ANF/BN	50	60/-	25.1	14
BC/Nano-clay	27	482/15	17.7	15
CNF/Amino-clay	50	205/9	10	16
CNF/Chitosan/MMT	10	135/14	3	17
CNF/PVA/MMT	70	302/-	3.7	18
Natural nacre	-	135/14.85~113.74	1.8	16
**PBO/MXene**	10	342.2/6.0	65.9	**This study**
	**20**	**416.7/9.1**	**97.3**	
	30	393.5/8.7	79.8	
	50	370.8/6.6	89.8	

-: no data presented in the cited literature.

Supplementary Table 4. Thermal diffusivity (α), Density (ρ), specific heat capacity (Cp) and thermal conductivity (TC) of the films.

Sample	In-plane α ($mm^2\ s^{-1}$)	Out-of-plane α ($mm^2\ s^{-1}$)	ρ ($g\ cm^{-3}$)	Cp ($J\ g^{-1}\ °C^{-1}$)	In-plane TC ($W\ m^{-1}\ K^{-1}$)	Out-of-plane TC ($W\ m^{-1}\ K^{-1}$)
PBO	15.49	0.158	1.460	1.131	25.6	0.261
PM10	21.13	0.078	1.523	1.030	33.1	0.122
PM20	25.53	0.062	1.660	0.996	42.2	0.103
PM30	26.82	0.065	1.699	0.911	41.5	0.101
PM50	23.51	0.119	1.487	0.798	28.0	0.141
PM70	18.32	0.135	1.607	0.718	21.1	0.156

Supplementary Table 5. Comparison of the multifunction of nacre-inspired PBO/MXene films with other polymer/2D inorganic nanosheet composites.

Materials	Loading (wt%)	Tensile strength (MPa)	TC (W m⁻¹ K⁻¹)	Combustion property	Electrical insulation (>1×10⁹ Ω cm)	Ref.
PVA/MXene/Fe_3O_4	40	21	2.9	-	No	19
CNF/MXene	60	114.4	14.9	Flame retardancy	-	20
PNF/MXene	70	125.1	5.82	Flame retardancy	No	21
CNF/ND/MXene	21.4	89.1	17.4	-	No	22
PVA/MXene	19.5	-	4.57	Flame retardancy	No	23
PBONF/GNS	50	192	100	Flame retardancy	No	7
NFC/GS	10	84.5	5.7	-	No	8
ANF/GF	20	188.5	48.2	-	No	9
CNF/rGO	8	314	29.5	-	No	24
CNF/GNP-g-L/D	5	111.8	9.36	-	Yes	25
Cellulose/BNNS	50	226	20.4	-	Yes	11
SiO_2-coated NFC/BNNS	7	166	10.9	-	Yes	12
ANF/BNNS	30	167	46.7	Flame retardancy	Yes	13
ANF/BN	50	60	64.1	-	Yes	14
**PBO/MXene**	**10**<br>**20**	**342.2**<br>**416.7**	**33.0**<br>**42.2**	**Flame retardancy**	**Yes**<br>**Yes**	**This Study**

-: no data presented in the cited literature.

Supplementary References

1. Lei, C., Zhang, Y., Liu, D., Wu, K. & Fu, Q. Metal-level robust, folding endurance, and highly temperature-stable MXene-based film with engineered aramid nanofiber for extreme-condition electromagnetic interference shielding applications. *ACS Appl. Mater. Interfaces* **12**, 26485-26495 (2020).
2. Hao, X. et al. Ultrastrong polyoxyzole nanofiber membranes for dendrite-proof and heat-resistant battery separators. *Nano Lett.* **16**, 2981-2987 (2016).
3. Cao, W. T. et al. Binary strengthening and toughening of MXene/cellulose nanofiber composite paper with nacre-inspired structure and superior electromagnetic interference shielding properties. *ACS Nano* **12**, 4583-4593 (2018).
4. Zhan, Z., Song, Q., Zhou, Z. & Lu, C. Ultrastrong and conductive MXene/cellulose nanofiber films enhanced by hierarchical nano-architecture and interfacial interaction for flexible electromagnetic interference shielding. *J. Mater. Chem. C* **7**, 9820-9829 (2019).
5. Zhou, Z., Song, Q., Huang, B., Feng, S. & Lu, C. Facile fabrication of densely packed Ti_3C_2 MXene/nanocellulose composite films for enhancing electromagnetic interference shielding and electro-/photothermal performance. *ACS Nano* **15**, 12405-12417 (2021).
6. Wan, S. et al. High-strength scalable MXene films through bridging-induced densification. *Science* **374**, 96-99 (2021).
7. Wang, Y., Xia, S., Li, H. & Wang, J. Unprecedentedly tough, folding-endurance, and multifunctional graphene-based artificial nacre with predesigned 3D nanofiber network as matrix. *Adv. Funct. Mater.* **29**, 1903876 (2019).
8. Song, N. et al. Layered nanofibrillated cellulose hybrid films as flexible lateral heat spreaders: The effect of graphene defect. *Carbon* **115**, 338-346 (2017).
9. Vu, M. C. et al. Nacre-inspired nanocomposite papers of graphene fluoride integrated 3D aramid nanofibers towards heat-dissipating applications. *Chem. Eng. J.* **429**, 132182 (2022).
10. Duan, J. et al. Bioinspired ternary artificial nacre nanocomposites based on reduced graphene oxide and nanofibrillar cellulose. *ACS Appl. Mater. Interfaces* **8**, 10545-10550 (2016).
11. Tu, H. et al. Superior strength and highly thermoconductive cellulose/boron nitride film by stretch-induced alignment. *J. Mater. Chem. A* **9**, 10304-10315 (2021).
12. Song, N. et al. Highly thermally conductive SiO_2-coated NFC/BNNS hybrid films with water resistance. *Composites, Part A* **143**, 106261 (2021).
13. Wu, K. et al. Highly thermoconductive, thermostable, and super-flexible film by engineering 1D rigid rod-like aramid nanofiber/2D boron nitride nanosheets. *Adv. Mater.* **32**, 1906939 (2020).
14. Xiao, G., Di, J., Li, H. & Wang, J. Highly thermally conductive, ductile biomimetic boron nitride/aramid nanofiber composite film. *Compos. Sci. Technol.* **189**, 108021 (2020).
15. Guan, Q.-F., Ling, Z.-C., Han, Z.-M., Yang, H.-B. & Yu, S.-H. Ultra-strong, ultra-tough, transparent, and sustainable nanocomposite films for plastic substitute. *Matter* **3**, 1308-1317 (2020).
16. Liu, Y., Yu, S.-H. & Bergström, L. Transparent and flexible nacre-like hybrid films of aminoclays and carboxylated cellulose nanofibrils. *Adv. Funct. Mater.* **28**, 1703277 (2018).

17. Yan, Y.-X., Yao, H.-B. & Yu, S.-H. Nacre-like ternary hybrid films with enhanced mechanical properties by interlocked nanofiber design. *Adv. Mater. Interfaces* **3**, 1600296 (2016).
18. Wang, J. F., Cheng, Q. F., Lin, L. & Jiang, L. Synergistic toughening of bioinspired poly(vinyl alcohol)-clay-nanofibrillar cellulose artificial nacre. *ACS Nano* **8**, 2739-2745 (2014).
19. Zhang, Y., Ruan, K. & Gu, J. Flexible sandwich-structured electromagnetic interference shielding nanocomposite films with excellent thermal conductivities. *Small* **17**, e2101951 (2021).
20. Jiao, E. et al. Ultrarobust MXene-based laminated paper with excellent thermal conductivity and flame retardancy. *Composites, Part A* **146**, 106417 (2021).
21. Wang, L. et al. Mechanically strong and folding-endurance $Ti_3C_2T_X$ MXene/PBO nanofiber films for efficient electromagnetic interference shielding and thermal management. *Carbon Energy* **4**, 200-210 (2022).
22. Jiao, E. et al. Nacre-like robust cellulose nanofibers/MXene films with high thermal conductivity and improved electrical insulation by nanodiamond. *J. Mater. Sci.* **57**, 2584-2596 (2022).
23. Jin, X. et al. Flame-retardant poly(vinyl alcohol)/MXene multilayered films with outstanding electromagnetic interference shielding and thermal conductive performances. *Chem. Eng. J.* **380**, 122475 (2020).
24. Shan, B., Xiong, Y., Li, Y., Yang, H. & Chen, Y. Sandwich structured RGO/CNF/RGO composite films for superior mechanical and thermally conductive properties. *Cellulose* **27**, 5055-5069 (2020).
25. Gu, T. et al. Highly thermally conductive, electrically insulated and flexible cellulose nanofiber-based composite films achieved via stereocomplex crystallites cross-linked graphene nanoplatelets. *Compos. Sci. Technol.* **230**, (2022).
